# Genome Sequences of Rare Human Enterovirus Genotypes Recovered from Clinical Respiratory Samples in Bern, Switzerland

**DOI:** 10.1128/mra.00276-22

**Published:** 2022-08-22

**Authors:** C. Grädel, N. R. Ireddy, M. C. Koch, C. Baumann, M. A. Terrazos Miani, M. T. Barbani, J. Steinlin-Schopfer, F. Suter-Riniker, S. L. Leib, A. Ramette

**Affiliations:** a Institute for Infectious Diseases, University of Bern, Bern, Switzerland; KU Leuven

## Abstract

We report on genomic sequences of human enteroviruses (EVs) that were identified in respiratory samples in Bern, Switzerland, in 2018 and 2019. Besides providing sequences for coxsackievirus A2, echovirus 11, and echovirus 30, we determined the sequences of rare EV-D68 and EV-C105 genotypes circulating in Switzerland.

## ANNOUNCEMENT

The viral genus *Enterovirus* belongs to the family *Picornaviridae*, which is associated with several human diseases (1). Some genotypes are predominantly isolated from respiratory samples (2, 3), e.g., the recently discovered species C genotypes enterovirus-C104 (EV-C104), EV-C105, and EV-C117 (4) or the well-known genotype EV-D68, which was linked to outbreaks of severe respiratory illness in children in summer 2014 in North America and cases of acute flaccid paralysis (5, 6). Various outbreaks of EV-D68 have since been reported worldwide (7–9).

A total of 145 respiratory samples from patients (average age of 3.5 years) who previously tested positive for EV or rhinovirus in 2018 or 2019 were screened for EV presence by PCR (ARGENE Rhino&EV/Cc R-GENE; bioMérieux, Geneva, Switzerland), immunofluorescence, and Sanger sequencing, as described previously (10, 11). Ethics approval was granted by the Swiss Ethics Committee (BASEC number Req-2018-00158). EVs were identified in six patients (4.1%), with two cases of EV-C105 and single cases of coxsackievirus A2 (CV-A2), echovirus 11, echovirus 30, and EV-D68. For one EV-C105 strain, EV-D68, and CV-A2, we further performed shotgun metatranscriptomic sequencing. Briefly, total RNA was extracted with the QIAamp viral RNA minikit according to the manufacturer's instructions (Qiagen, Switzerland). Next, a 20-μL cDNA synthesis reaction was prepared. First, a mixture of 1 μL random hexamers (Thermo Fisher Scientific), 1 μL 10 mM deoxynucleoside triphosphate (dNTP) mix (New England Biolabs, Ipswich, MA, USA), 1 μL nuclease-free water (NFW), and 8 μL RNA extract was heated at 65°C for 5 min. Second, a preparation of 4 μL 5× SuperScript IV reaction buffer (Invitrogen, Carlsbad, CA), 2 μL dithiothreitol (DTT), 200 units SuperScript IV reverse transcriptase (Invitrogen), 40 units RNaseOUT (Invitrogen), and 1 μL NFW was made, and the two preparations were mixed and incubated at 25°C for 10 min, at 50°C for 10 min, and at 80°C for 10 min. The Illumina Nextera DNA Flex kit (paired-end reads, 300 cycles) was used for library preparation, followed by sequencing on a MiSeq benchtop sequencer, as described previously (12). Read trimming and quality filtering (fastp v0.20.0 [13]) were followed by genome assembly (SPAdes v3.13.2 [14]) and multiple sequence alignments (MAFFT v7.313 [15]). All software and scripts were used with default values. We thus recovered 96.7% of the genome sequence of EVCG-05-BE (EV-C105), 99.7% of EVCG-06-BE (EV-D68), and 85.7% of EVCG-03-BE (CV-A2), compared to their closest reference sequences (Table 1).

**TABLE 1:** Sample and sequencing details

Sequence name	Taxonomy	Collection date	Patient age (yr)	Type of sequencing^*a*^	Sequence length (nucleotides)	GenBank accession no.	SRA accession no.	GenBank accession no. for closest sequence^*b*^	Identity to closest sequence (no. of identical nucleotides/total no. of nucleotides [%])^*b*^	Total no. of reads^*c*^	No. of reads mapping to EV (% of total)
EVCG-01-BE	Echovirus E11	December 2018	<1	VP1	322	OW121671		MN121654.1	312/322 (96.9)	NA	NA
EVCG-02-BE	Echovirus E30	December 2018	<1	VP1	325	OW122598		MK815529.1	325/325 (100)	NA	NA
EVCG-03-BE	CV-A2	June 2018	<1	MTT	7,372	OW122596	ERR9235688	MT350223.1	6,271/7,315 (85.7)	4,560,107	10,681 (0.23)
EVCG-04-BE	EV-C105	February 2019	70–75	VP1	275	OW122599		KX901639.1	267/275 (97.1)	NA	NA
EVCG-05-BE	EV-C105	March 2018	<5	MTT	7,288	OW122597	ERR9235689	KX276189.1	6,971/7,208 (96.7)	3,917,728	502 (0.013)
EVCG-06-BE	EV-D68	September 2018	<1	MTT	6,898	OW122595	ERR9235690	MK419059.1	6,881/6,898 (99.7)	2,526,310	1,360 (0.054)

a VP1 gene amplicons were sequenced via Sanger sequencing; genome sequences were obtained by read assembly after shotgun metatranscriptomic (MTT) sequencing.

b The closest sequence was that associated with the highest score via the NCBI BLASTn algorithm.

c From Illumina MiSeq paired-end sequencing (300 cycles). NA, not applicable.

**FIG 1:**
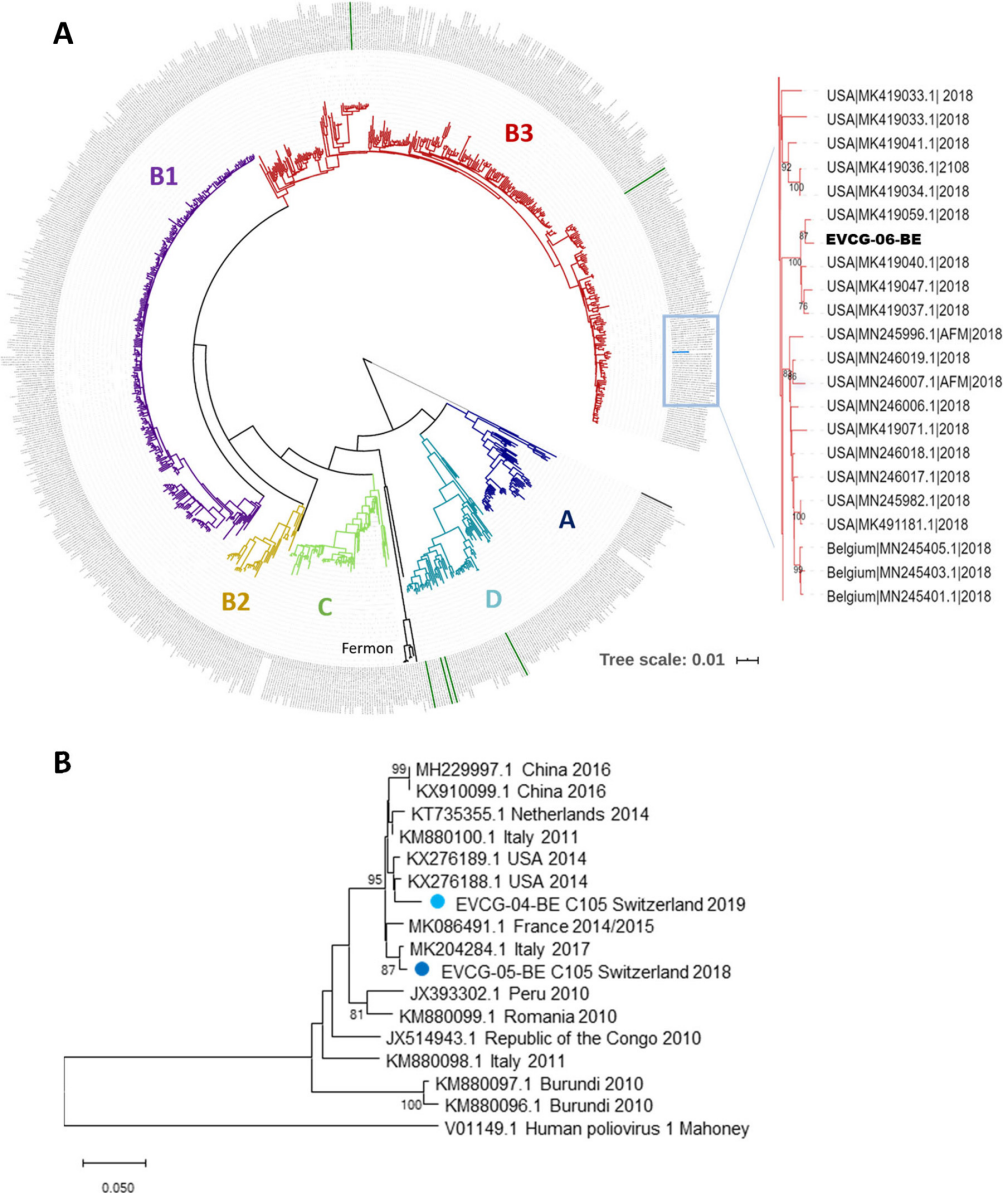
(A) Phylogenetic tree of the EV-D68 genome sequence (EVCG-06-BE) from Bern, Switzerland, with all available EV-D68 genome sequences. The inset on the right indicates phylogenetic relationships in the close neighborhood of the reported EV-D68 sequence (bootstrap support cutoff, ≥70%). Labels consist of isolate's country of origin, accession number, followed by year of isolation separated by vertical bars. Labels of other Swiss EV-D68 genomes retrieved from GenBank (MN245406 to MN245411) are colored in dark green in the circular phylogenetic tree. (B) VP1-based neighbor-joining phylogenetic tree of EV-C105 sequences. The two strains found in Bern, Switzerland (blue circles), were compared with all available EV-C105 sequences in GenBank on the basis of their partial VP1 sequences. Poliovirus 1 was added as an outgroup. Labels consist of sequence accession number, isolate's country of origin, followed by year of isolation separated by spaces. The scale bars represent the number of base substitutions per site. Bootstrap values were set to 1,000 replicates for both phylogenies. Phylogenetic trees were created using MEGA X (23) and further edited using the iTOL platform (https://itol.embl.de).

Sequence analysis showed that the Bern EV-D68 case clustered with subclade B3 sequences isolated from the United States (Fig. 1A), one of the most commonly reported EV-68 clades circulating worldwide in 2018 (16, 17). Analysis of all EV-C105 sequences (VP1) in GenBank showed that the two identified Bern EV-C105 isolates may correspond to different circulating strains (Fig. 1B). Only a few genome sequences of this globally circulating genotype (4, 18–20) are available to date (Fig. 1B).

Here, we report the first near-complete sequence of the EV-D68 genotype in Bern, Switzerland. Outbreaks of EV-D68 were reported widely in Europe in 2018 (16, 21, 22). Together with six published sequences reported in 2018 in Basel, Switzerland (17), our study sheds light on the recent circulation of EV-D68 in Switzerland, a country with few data on this genotype to date. Furthermore, we present the first two sequences of the rarely reported EV-C105 genotype in Switzerland.

### Data availability.

The consensus genome sequences and associated raw data were deposited in the European Nucleotide Archive (ENA) under BioProject accession number PRJEB51320; the corresponding accession numbers for samples are provided in Table 1.

## References

[1] World Health Organization. 2015. Enterovirus surveillance guidelines: guidelines for enterovirus surveillance in support of the Polio Eradication Initiative. World Health Organization Regional Office for Europe, Copenhagen, Denmark. http://www.euro.who.int/__data/assets/pdf_file/0020/272810/EnterovirusSurveillanceGuidelines.pdf.

[2] Royston L, Tapparel C. 2016. Rhinoviruses and respiratory enteroviruses: not as simple as ABC. Viruses 8:16. doi:10.3390/v8010016. PMC4728576 26761027

[3] Tapparel C, Siegrist F, Petty TJ, Kaiser L. 2013. Picornavirus and enterovirus diversity with associated human diseases. Infect Genet Evol 14:282–293. doi:10.1016/j.meegid.2012.10.016. 23201849

[4] Van Leer-Buter CC, Poelman R, Borger R, Niesters HGM. 2016. Newly identified enterovirus C genotypes, identified in the Netherlands through routine sequencing of all enteroviruses detected in clinical materials from 2008 to 2015. J Clin Microbiol 54:2306–2314. doi:10.1128/JCM.00207-16. 27358467 PMC5005491

[5] Midgley CM, Jackson MA, Selvarangan R, Turabelidze G, Obringer E, Johnson D, Giles BL, Patel A, Echols F, Oberste MS, Nix WA, Watson JT, Gerber SI. 2014. Severe respiratory illness associated with enterovirus D68: Missouri and Illinois, 2014. MMWR Morb Mortal Wkly Rep 63:798–799. 25211545 PMC4584696

[6] Sejvar JJ, Lopez AS, Cortese MM, Leshem E, Pastula DM, Miller L, Glaser C, Kambhampati A, Shioda K, Aliabadi N, Fischer M, Gregoricus N, Lanciotti R, Nix WA, Sakthivel SK, Schmid DS, Seward JF, Tong S, Oberste MS, Pallansch M, Feikin D. 2016. Acute flaccid myelitis in the United States, August–December 2014: results of nationwide surveillance. Clin Infect Dis 63:737–745. doi:10.1093/cid/ciw372. 27318332 PMC5709818

[7] Poelman R, Schuffenecker I, Van Leer-Buter C, Josset L, Niesters HGM, Lina B, ESCV-ECDC EV-D68 Study Group. 2015. European surveillance for enterovirus D68 during the emerging North-American outbreak in 2014. J Clin Virol 71:1–9. doi:10.1016/j.jcv.2015.07.296. 26364237

[8] Funakoshi Y, Ito K, Morino S, Kinoshita K, Morikawa Y, Kono T, Doan YH, Shimizu H, Hanaoka N, Konagaya M, Fujimoto T, Suzuki A, Chiba T, Akiba T, Tomaru Y, Watanabe K, Shimizu N, Horikoshi Y. 2019. Enterovirus D68 respiratory infection in a children's hospital in Japan in 2015. Pediatr Int 61:768–776. doi:10.1111/ped.13903. 31136073 PMC7167638

[9] Holm-Hansen CC, Midgley SE, Fischer TK. 2016. Global emergence of enterovirus D68: a systematic review. Lancet Infect Dis 16:e64–e75. doi:10.1016/S1473-3099(15)00543-5. 26929196

[10] Grädel C, Terrazos Miani MA, Barbani MT, Leib SL, Suter-Riniker F, Ramette A. 2019. Rapid and cost-efficient enterovirus genotyping from clinical samples using Flongle flow cells. Genes (Basel) 10:659. doi:10.3390/genes10090659. PMC6770998 31470607

[11] Barbani MT, Gorgievski-Hrisoho M. 2009. Rapid detection of respiratory picornaviruses in nasopharyngeal aspirates by immunofluorescence assay. J Clin Virol 45:245–248. doi:10.1016/j.jcv.2009.05.008. 19502108 PMC7172351

[12] Grädel C, Terrazos Miani MA, Baumann C, Barbani MT, Neuenschwander S, Leib SL, Suter-Riniker F, Ramette A. 2020. Whole-genome sequencing of human enteroviruses from clinical samples by Nanopore direct RNA sequencing. Viruses 12:841. doi:10.3390/v12080841. PMC7472277 32752120

[13] Chen S, Zhou Y, Chen Y, Gu J. 2018. fastp: an ultra-fast all-in-one FASTQ preprocessor. Bioinformatics 34:i884–i890. 30423086 10.1093/bioinformatics/bty560 PMC6129281

[14] Bankevich A, Nurk S, Antipov D, Gurevich AA, Dvorkin M, Kulikov AS, Lesin VM, Nikolenko SI, Pham S, Prjibelski AD, Pyshkin AV, Sirotkin AV, Vyahhi N, Tesler G, Alekseyev MA, Pevzner PA. 2012. SPAdes: a new genome assembly algorithm and its applications to single-cell sequencing. J Comput Biol 19:455–477. doi:10.1089/cmb.2012.0021. 22506599 PMC3342519

[15] Katoh K, Misawa K, Kuma K-I, Miyata T. 2002. MAFFT: a novel method for rapid multiple sequence alignment based on fast Fourier transform. Nucleic Acids Res 30:3059–3066. doi:10.1093/nar/gkf436. 12136088 PMC135756

[16] Bal A, Sabatier M, Wirth T, Coste-Burel M, Lazrek M, Stefic K, Brengel-Pesce K, Morfin F, Lina B, Schuffenecker I, Josset L. 2019. Emergence of enterovirus D68 clade D1, France, August to November 2018. Euro Surveill 24:1800699. doi:10.2807/1560-7917.ES.2019.24.3.1800699. PMC6344839 30670143

[17] Hodcroft EB, Dyrdak R, Andrés C, Egli A, Reist J, García Martínez de Artola D, Alcoba-Flórez J, Niesters HGM, Antón A, Poelman R, Reynders M, Wollants E, Neher RA, Albert J. 2022. Evolution, geographic spreading, and demographic distribution of enterovirus D68. PLoS Pathog 18:e1010515. doi:10.1371/journal.ppat.1010515. 35639811 PMC9212145

[18] Tapparel C, Junier T, Gerlach D, Van-Belle S, Turin L, Cordey S, Mühlemann K, Regamey N, Aubert J-D, Soccal PM, Eigenmann P, Zdobnov E, Kaiser L. 2009. New respiratory enterovirus and recombinant rhinoviruses among circulating picornaviruses. Emerg Infect Dis 15:719–726. doi:10.3201/eid1505.081286. 19402957 PMC2687021

[19] Lukashev AN, Drexler JF, Kotova VO, Amjaga EN, Reznik VI, Gmyl AP, Grard G, Taty Taty R, Trotsenko OE, Leroy EM, Drosten C. 2012. Novel serotypes 105 and 116 are members of distinct subgroups of human enterovirus C. J Gen Virol 93:2357–2362. doi:10.1099/vir.0.043216-0. 22894922

[20] Daleno C, Piralla A, Scala A, Baldanti F, Usonis V, Principi N, Esposito S. 2012. Complete genome sequence of a novel human enterovirus C (HEV-C117) identified in a child with community-acquired pneumonia. J Virol 86:10888–10889. doi:10.1128/JVI.01721-12. 22966184 PMC3457295

[21] Pellegrinelli L, Giardina F, Lunghi G, Uceda Renteria SC, Greco L, Fratini A, Galli C, Piralla A, Binda S, Pariani E, Baldanti F. 2019. Emergence of divergent enterovirus (EV) D68 sub-clade D1 strains, northern Italy, September to October 2018. Euro Surveill 24:1900090. doi:10.2807/1560-7917.ES.2018.24.7.1900090. PMC6381661 30782269

[22] Cottrell S, Moore C, Perry M, Hilvers E, Williams C, Shankar AG. 2018. Prospective enterovirus D68 (EV-D68) surveillance from September 2015 to November 2018 indicates a current wave of activity in Wales. Euro Surveill 23:1800578. doi:10.2807/1560-7917.ES.2018.23.46.1800578. PMC6247461 30458915

[23] Kumar S, Stecher G, Li M, Knyaz C, Tamura K. 2018. MEGA X: Molecular Evolutionary Genetics Analysis across computing platforms. Mol Biol Evol 35:1547–1549. doi:10.1093/molbev/msy096. 29722887 PMC5967553

